# Altered cellular localization and hemichannel activities of KID syndrome associated connexin26 I30N and D50Y mutations

**DOI:** 10.1186/s12860-016-0081-0

**Published:** 2016-02-02

**Authors:** Hande Aypek, Veysel Bay, Gülistan Meşe

**Affiliations:** Department of Molecular Biology and Genetics, Izmir Institute of Technology, Urla, Izmir Turkey

**Keywords:** Connexin26, Hemichannels, Keratitis-ichthyosis-deafness, Intracellular calcium

## Abstract

**Background:**

Gap junctions facilitate exchange of small molecules between adjacent cells, serving a crucial function for the maintenance of cellular homeostasis. Mutations in connexins, the basic unit of gap junctions, are associated with several human hereditary disorders. For example, mutations in connexin26 (Cx26) cause both non-syndromic deafness and syndromic deafness associated with skin abnormalities such as keratitis-ichthyosis-deafness (KID) syndrome. These mutations can alter the formation and function of gap junction channels through different mechanisms, and in turn interfere with various cellular processes leading to distinct disorders. The KID associated Cx26 mutations were mostly shown to result in elevated hemichannel activities. However, the effects of these aberrant hemichannels on cellular processes are recently being deciphered. Here, we assessed the effect of two Cx26 mutations associated with KID syndrome, Cx26I30N and D50Y, on protein biosynthesis and channel function in N2A and HeLa cells.

**Results:**

Immunostaining experiments showed that Cx26I30N and D50Y failed to form gap junction plaques at cell-cell contact sites. Further, these mutations resulted in the retention of Cx26 protein in the Golgi apparatus. Examination of hemichannel function by fluorescent dye uptake assays revealed that cells with Cx26I30N and D50Y mutations had increased dye uptake compared to Cx26WT (wild-type) containing cells, indicating abnormal hemichannel activities. Cells with mutant proteins had elevated intracellular calcium levels compared to Cx26WT transfected cells, which were abolished by a hemichannel blocker, carbenoxolone (CBX), as measured by Fluo-3 AM loading and flow cytometry.

**Conclusions:**

Here, we demonstrated that Cx26I30N and D50Y mutations resulted in the formation of aberrant hemichannels that might result in elevated intracellular calcium levels, a process which may contribute to the hyperproliferative epidermal phenotypes of KID syndrome.

**Electronic supplementary material:**

The online version of this article (doi:10.1186/s12860-016-0081-0) contains supplementary material, which is available to authorized users.

## Background

Gap junctions facilitate the intercellular communication between adjacent cells by allowing the exchange of small molecules that play roles in the regulation of many cellular events including proliferation, differentiation and cellular homeostasis [1, 2]. Gap junction biosynthesis starts in the ER-Golgi network by the oligomerization of six connexin subunits into hemichannels, known as connexons. Then, these connexons are transported to the plasma membrane where they can either align with other connexons from neighboring cells to complete the formation of intercellular channels, or function individually on the plasma membrane as hemichannels that can mediate the exchange of materials between the cell interior and the extracellular environment [3–8].

Gap junction channels and hemichannels are important modulators of tissue homeostasis as evidenced by the association of mutations in connexin genes with several hereditary disorders. For example, mutations in connexin 26 (Cx26), a member of the connexin gene family, are the leading cause of non-syndromic hearing loss (NSHL) [9]. To date, more than 100 NSHL mutations throughout the Cx26 gene (GJB2) have been identified, and functional characterization of these mutations suggested that the majority are loss-of-function mutations that can lead to protein truncation, altered trafficking, misfolding of Cx26 protein or altered channel permeability [3, 10–12]. In addition to NSHL, Cx26 mutations were linked to syndromic deafness associated with various skin pathologies including keratitis-ichthyosis-deafness (KID) syndrome, palmoplantar keratoderma (PPK) and Vohwinkel syndrome (VS) [13]. Generation of diverse epidermal phenotypes during aforementioned skin disorders driven by different Cx26 mutations imply that associated mutations may have unique properties affecting distinct cellular machinery [3, 10, 14].

KID syndrome is a rare congenital genetic disorder with phenotypes of thickening of the skin, keratisis, scaly skin (ichthyosis) and deafness [15, 16]. The Cx26A40V was the first KID syndrome associated mutation that was shown to cause increased hemichannel activities and cell death in mRNA injected *Xenopus* oocytes [17]. Other studies on different KID syndrome mutations also suggested the involvement of altered hemichannel activities and cell death in both Xenopus oocytes and mammalian cell lines as a common mechanism for this disorder [18–22]. However, the molecular mechanisms that lead to cell death, and skin phenotypes due to active hemichannels are poorly understood. For that, we characterized two more KID syndrome mutations, Cx26I30N and D50Y, in order to understand whether they affect the protein biosynthetic pathway, hemichannel activities similar to previously characterized KID syndrome mutations and intracellular calcium levels that play essential roles in cellular processes, especially in keratinocyte proliferation, differentiation and migration [23–25].

## Results

### Protein localization of Cx26I30N and D50Y mutant proteins

**Fig. 1:**
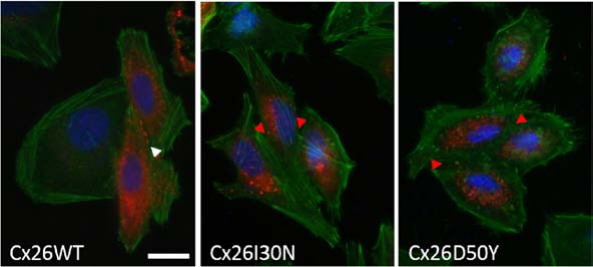
Effect of Cx26I30N and D50Y mutations on protein expression and localization. Merged images of Cx26WT, I30N and D50Y transfected HeLa cells that were co-stained with phalloidin for actin (*green*) and Cx26 antibody (*red*). *Blue* is for DAPI staining of the nucleus. *White arrow* head shows the gap junction plaques formed between adjacent Cx26WT expressing cells. Red arrow heads point the cell-to-cell contact sites between neighboring cells of Cx26I30N and D50Y. Cells with only green and blue signals indicate untransfected cells. Scale bar 10 μm

To examine the effects of Cx26I30N and D50Y KID syndrome associated mutations on protein synthesis and localization, gap junctional communication deficient cell line, HeLa, were transiently transfected with pIRES2EGFP2 Cx26WT (wild-type), I30N and D50Y constructs. 24 h after transfection, the protein synthesis and localization was determined by immunofluorescent staining of transfected cells (Fig. 1). Immunofluorescent staining with Cx26 specific antibody demonstrated that cells expressing Cx26WT, I30N and D50Y constructs were able to synthesize Cx26 proteins (Fig. 1). Further, cells transfected with Cx26WT targeted proteins to the plasma membrane where they formed gap junction plaques at the cell-to-cell junctions between adjacent cells (Fig. 1, white arrow head). On the other hand, in spite of positive protein synthesis in cells with Cx26I30N or D50Y constructs, no gap junctional plaques were observed between neighboring cells expressing mutant constructs (Fig. 1, red arrow heads). This suggested that Cx26I30N and D50Y proteins failed to form gap junction plaques between adjacent cells.

### Effect of Cx26I30N and D50Y mutations on the Cx26 protein trafficking

**Fig. 2:**
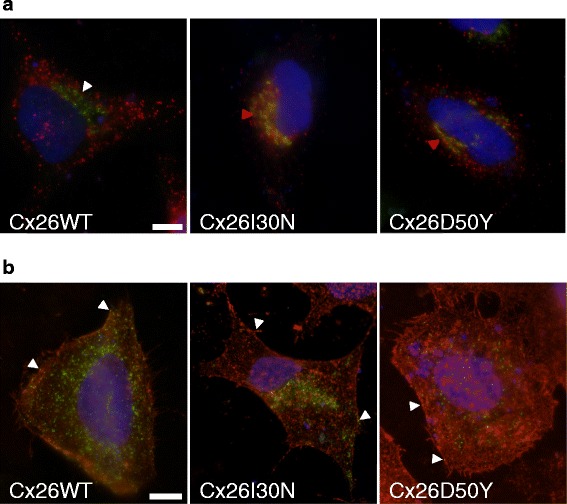
Effect of Cx26I30N and D50Y mutations on protein trafficking. **a** Merged images of Cx26WT, I30N and D50Y transfected cells that were co-stained with Cx26 (*red*) and the Golgi apparatus marker, golgin-97 (*green*) antibodies. Blue is for DAPI staining of the nucleus. Arrow heads point the location of the Golgi apparatus. Yellow signals in Cx26I30N and D50Y images show the colocalization of Cx26 and golgin-97. Scale bar 10 μm. **b** Merged images of Cx26WT, I30N and D50Y transfected cells that were co-stained with Cx26 (*green*) antibody and Rhodamine labeled wheat germ agglutinin (WGA). Blue is for DAPI staining of the nucleus. Yellow signals shown by arrow heads point out the colocalization of Cx26 and WGA. Scale bar 10 μm

In order to determine the location of Cx26 proteins within cells, co-labelling of Cx26 protein with golgin-97, a Golgi apparatus marker, or Cx26 protein with Wheat germ agglutinin (WGA), for the plasma membrane staining, were performed (Fig. 2). As observed in images in Fig. 2a, there was no overlap between Cx26 and golgin-97 protein signals in Cx26WT expressing cells while Cx26I30N or D50Y mutant proteins were widely co-localized with golgin-97 in transfected cells as evidenced by the presence of yellow signals (Fig. 2a, red arrow heads). Analysis of Costes’ colocalization values through image processing revealed that 2 out of 25 (8 %) images for Cx26WT had significant colocalization for Cx26 protein and golgin-97 (p > 95 %). On the other hand, 19 out of 33 images (58 %) for Cx26I30N and 13 out of 26 (50 %) for Cx26D50Y had significant co-localization, ratios that were both significantly (*p* < 0.01) higher compared to WT images (Table 1). Furthermore, the membrane localization of Cx26WT, I30N and D50Y, that was verified by Cx26 and Rhodamine labeled WGA co-staining suggested that a fraction of proteins in all conditions were localized to the plasma membrane (Fig. 2b, arrow heads). These suggested that mutant proteins can be found both in the Golgi apparatus and the plasma membrane.

**Table 1:** Analysis of colocalization between Cx26 and golgin-97, the Golgi apparatus marker

	Cells analyzed (n)	Colocalization (n)	Ratio (%)
Cx26WT	25	2	8^a^
Cx26I30N	33	19	58^b^
Cx26D50Y	26	13	50^b^

^a,b^Different letters indicate statistically significant differences among groups

### Altered hemichannel activities of Cx26I30N and D50Y channels

**Fig. 3:**
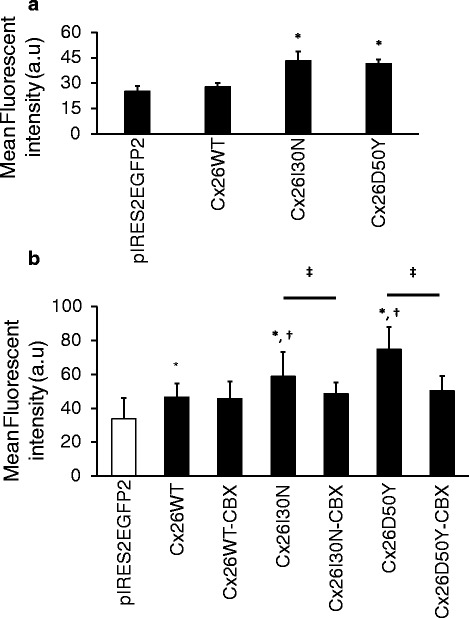
Fluorescent dye uptake in Cx26WT, I30N and D50Y transfected cells. **a** Comparison of fluorescent dye intensities in transfected cells under physiological calcium concentration. Cx26I30N and D50Y were compared with both negative control pIRES2EGFP2 and Cx26WT (*, *p* < 0.01). **b** Comparison of fluorescent dye intensities in transfected cells in divalent free medium and in the presence of 100 μM CBX, a hemichannel blocker. Cx26WT, I30N and D50Y were compared with negative control pIRES2EGFP2 (*, *p* < 0.01); Cx26I30N and D50Y were compared with Cx26WT (†, *p* < 0.01) and comparison between samples treated with or without CBX was performed (‡, *p* < 0.01)

Cx26 mutations associated with KID syndrome have been shown to cause elevated hemichannel activities [19, 21, 22]. Here, we determined the ability of Cx26I30N mutation to form aberrant hemichannels in addition to D50Y mutation that was previously shown to result in elevated membrane currents in *Xenopus* oocytes by electrophysiological measurements [26] in mammalian cell lines with fluorescent dye uptake assay using Neurobiotin (NB) (Fig. 3). Under physiological calcium concentrations, Cx26WT hemichannels had 10 % increase in the mean fluorescent intensity of NB in cells compared to negative control. On the other hand, Cx26I30N and D50Y expressing cells had 1.5 fold increase in NB uptake with respect to WT containing cells (*n* = 15 images, *p* < 0.01) (Fig. 3a), suggesting an aberrant hemichannel activities. To further verify the formation of abnormal hemichannels on the plasma membrane, dye uptake studies were performed under divalent free conditions and in the presence of a hemichannel blocker, carbenoxolone (CBX) (Fig. 3b). Analysis of NB fluorescent intensity in EGFP positive cells suggested that the fluorescent intensity of Cx26WT cells was 1.3 fold (*n* = 50 images, *p* < 0.01) higher than control cells and for I30N and D50Y cells the fluorescent intensities increased by 1.3 (*n* = 60 images, *p* < 0.01) and 1.6 fold (*n* = 49 images, *p* < 0.01) compared to Cx26WT cells, respectively (Fig. 3b). To determine if the uptake of NB into the cells were mediated by Cx26I30N and D50Y abnormal hemichannels, cells were initially treated with a hemichannel blocker, carbenoxolone (CBX) before NB application. Treatment of cells with 100 μM CBX for 20 min resulted in a 17 % and 35 % (both, *p* < 0.01) reduction in the levels of mean fluorescent intensities in Cx26I30N and D50Y expressing cells compared to their CBX absent counterparts, respectively. These suggested that the increase in the uptake of NB into cells were mediated by aberrant hemichannels formed from Cx26I30N and D50Y.

### Effect of Cx26I30N and D50Y mutations on intracellular calcium signals

**Fig. 4:**
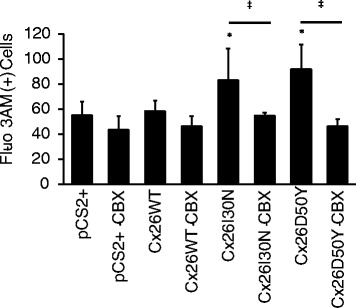
Effect of Cx26 mutations on intracellular calcium levels. Intracellular calcium concentration determined by a calcium indicator (Fluo-3 AM) using flow cytometry was compared among pCS2+, Cx26WT, I30N and D50Y containing cells (*, *p* < 0.01). Comparison between 100 μM CBX treated and non-treated samples was also performed (‡, *p* < 0.01)

Calcium signals are essential for the maintenance of epidermal homeostasis [27–29]. Therefore, we next wanted to investigate whether Cx26I30N and D50Y mutations have any effect on internal calcium content by using a calcium indicator, Fluo-3 AM, and flow cytometry (Fig. 4). Comparison of Fluo-3 AM signals in pCS2+, Cx26WT, I30N and D50Y transfected cells demonstrated that intracellular calcium content in both I30N and D50Y transfected cells were elevated by 1.4 (*p* < 0.01) and 1.6 fold (*p* < 0.01) compared to cells with pCS2+ and Cx26WT clones, respectively (Fig. 4). This increase in intracellular calcium content was reduced by 34 % and 50 % (both, *p* < 0.01) with the treatment of 100 μM CBX in Cx26I30N and D50Y containing cells, respectively, suggesting the involvement of aberrant hemichannels in the elevation of intracellular calcium concentration (Fig. 4).

## Discussion

Characterization of Cx26 mutations associated with KID syndrome will improve the understanding of both Cx26 function in normal epidermal homeostasis and generation of disease phenotypes in affected individuals. Examination of several Cx26 mutations suggested that KID syndrome mutations lead to the formation of active hemichannels on the plasma membrane that might result in uncontrolled exchange of molecules between the cytosol and the extracellular environment, influencing the cellular/tissue homeostasis [19, 30, 31]. Here, we characterized two additional KID syndrome mutations, Cx26I30N and D50Y, in order to understand their effect on the protein biosynthetic pathway, hemichannel activity and intracellular calcium levels in communication deficient cell lines. We demonstrated that both mutants failed to form gap junction plaques at cell-to-cell contact sites between neighboring cells in contrast to Cx26WT proteins even though a fraction of mutant proteins was targeted to the plasma membrane. Moreover, cells with mutant constructs had increased uptake of neurobiotin fluorescent dye and elevated intracellular calcium levels compared to cells with Cx26WT which were abolished by carbenoxolone, a hemichannel blocker. These observations provided support for the contribution of aberrant hemichannels and the involvement of altered calcium signals in KID syndrome [18, 32].

Mutations in the Cx26 gene cause both non-syndromic and syndromic deafness with skin pathologies. Each mutation is unique in terms of defects they cause, that is, mutations leading to various skin disorders result in distinct molecular abnormalities within cells at different levels from protein synthesis to the formation and the function of gap junction channels [3, 12]. Mutations associated with KID syndrome, for example, were shown to mostly cause abnormal hemichannel activity leading to altered regulation of molecular exchange through the plasma membrane [19–22, 33]. Similar to many characterized KID syndrome mutations, Cx26I30N and D50Y also resulted in increased fluorescent dye uptake from the external environment, suggesting the involvement of aberrant hemichannel activities for these mutations.

Disease associated Cx26 mutations lead to different molecular abnormalities that can interfere with the cellular homeostasis [3]. Included among these are alteration in trafficking of proteins to the plasma membrane, where they could prevent the formation of gap junction plaques at cell-to-cell contact sites. Abnormal membrane trafficking might result in the accumulation of mutant proteins within the subcellular structures including the ER, the ER-Golgi intermediate compartment or the Golgi apparatus [34–37]. In this study, Cx26I30N and D50Y mutations were observed to abolish the formation of gap junction plaques between adjacent cells. Although mutant proteins were observed on the plasma membrane, they were also widely accumulated in the Golgi apparatus. How mutations in isoleucine 30 (I30) and aspartic acid at position 50 (D50) affect the localization and function of Cx26 is not known. I30 is found in the first transmembrane domain (TM1) that was shown to be important for oligomerization and channel function [1, 38]. The mutation of a nonpolar residue into a polar amino acid (asparagine) might affect the structure of the TM1 as asparagine can form hydrogen bonds with other residues that can affect the organization and stability of this domain. The inability of I30N mutation to form gap junction channels suggest that this amino acid might influence the docking of hemichannels in the extracellular space during the formation of gap junction channels. The first extracellular loop (EC1) was shown to be involved in connexon formation, docking of connexons at the extracellular space and gap junction channel function [1, 39]. D50 in EC1 is highly conserved across species and across the connexin family [16]. The mutation of this charged amino acid might alter the organization of the EC1, affecting the interactions of connexons in the ESC as they are forming the gap junction channels [22, 30].

The maintenance of calcium homeostasis is crucial for proper functioning of keratinocytes and the epidermis. Calcium plays roles in the regulation of keratinocyte proliferation, differentiation and cellular adhesion processes [29, 40]. Furthermore, there is a calcium gradient across the epidermis with the lowest concentration in the stratum basale and the highest in the stratum granulosum. Maintenance of this gradient and intracellular calcium levels are indispensable for epidermal cells as abnormalities in these processes have been linked to a variety of skin disorders [41–43]. Here, Cx26I30N and D50Y mutations were shown to result in the elevation of intracellular calcium in transfected cells compared to Cx26WT cells. An increase in the intracellular calcium levels was also observed for other Cx26 KID syndrome associated mutations [18, 32]. Terrinoni et al. demonstrated that cells with Cx26G11E and D50N mutations have an increased amount of intracellular calcium 24 h post transfection [18]. Moreover, Garcia et al. observed a significant increase in the intracellular calcium levels in cells co-expressing Cx43 and KID syndrome associated Cx26 mutant constructs compared to Cx43 alone cells [32]. Thus, deregulation of intracellular calcium mechanisms by aberrant hemichannel activities may perturb several calcium dependent cellular processes to contribute to the development of skin phenotypes.

## Conclusion

Formation of aberrant hemichannels due to Cx26I30N and D50Y mutations provided further support for the involvement of abnormal hemichannel activities as a common mechanism for KID syndrome. Uncontrolled transport of signaling molecules such as ATP or calcium through these channels may alter keratinocyte growth, proliferation and/or differentiation that could play role in the generation of dermatological anomalies in KID patients. However, the molecular mechanisms altered by aberrant Cx26 hemichannels affecting keratinocyte proliferation, differentiation and migration remain to be elusive.

## Methods

### Site-directed mutagenesis and construction of Cx26 mutant clones

Cx26I30N and D50Y missense point mutations were generated from human wild-type Cx26 cDNA in pBlueScript II (courtesy of Prof. Dr. Thomas W. White, Stony Brook University, Stony Brook, NY, USA) by using site-directed mutagenesis. PCR products were cloned into pBlueScript II cloning vector and location and insertion of mutations were verified by sequencing (Macrogen Europe, the Netherlands). Then, Cx26WT, I30N and D50Y cDNAs were subcloned into pIRES2EGFP2 (Clontech Laboratories, USA) and pCS2+ mammalian expression vectors for immunostaining and functional studies.

### Cell culture

Gap junctional communication deficient cell lines, HeLa (human cervical cell line) and neuro-2A (N2A, mouse neuroblastoma cell line) (purchased from ATCC, USA), were maintained in Dulbecco’s Modified Eagle Medium (DMEM, Thermo Scientific HyClone, USA) supplemented with 10 % fetal bovine serum (FBS) (Biological Industries, Israel) and 1 % penicillin/streptomycin (GIBCO, USA) in a humidified chamber with 5 % CO_2_ and 37 °C. For experiments, cells were plated in 6 well plates so that they would reach 70-80 % confluence on the day of transfection with Lipofectamine 2000 reagent (Invitrogen, USA). Cells were transfected with 1:2 DNA to Lipofectamine 2000 ratio following the manufacturer’s protocol and 3.2 mM CaCI_2_ was added to prevent the cell death due to the activities of prospective hemichannels. Cells were used within 24–48 h after transfection.

### Immunofluorescent stainings

HeLa cells (2,5x10^5^) were grown over glass coverslips in 6 well plates for immunofluorescent staining experiments. 24 h post transfection, cells were washed with PBS and then fixed with 4 % paraformaldehyde (PFA) for 20 min, permeabilized with 0.1 % Triton-X 100 for 15 min and blocked with 3 % bovine serum albumin (BSA) for 1 h at room temperature (RT). Cells were then incubated with a 1:500 dilution of a polyclonal rabbit antibody against Cx26 (Invitrogen, USA) for 1 h at RT followed by an application of a 1:200 dilution of Alexa555-conjugated goat anti-rabbit antibody (Invitrogen, USA), 1:200 dilution of Alexa Fluor 488 conjugated Phalloidin (Invitrogen, USA) and 1 μM DAPI for 45 min at RT in dark. For co-immunofluorescent staining of golgin-97, a marker for the Golgi apparatus, and Cx26 proteins, cells were incubated with primary antibodies (rabbit anti-Cx26 antibody (1:500) and mouse anti-golgin-97 (1:1000) (Invitrogen, USA)) for 1 h at room temperature. Then, secondary antibodies (Alexa555-conjugated goat anti-rabbit (Invitrogen, USA) and Alexa488-conjugated goat anti-mouse secondary antibody (1:200) (Invitrogen, USA) and 1 μM DAPI were applied to cells for 45 min at RT in dark. After washing with PBS, coverslips were dipped in distilled water, dried and mounted on glass slides. Staining was verified under fluorescence microscope (IX83, Olympus, Japan) with x40 or x100 oil-immersion objective and images were taken with a CCD digital camera.

For co-labeling of Rhodamine labeled Wheat Germ Agglutinin (WGA) and Cx26, after washing cells with PBS twice, 1:200 dilution of Rhodamine labeled WGA (5 mg/ml, Vector Labs, USA) was applied to cells for 30 min at 4 °C. Following the wash with PBS, cells were fixed and immunostained with Cx26 as explained above. Staining was verified under fluorescent microscope (IX83, Olympus, Japan) with x100 oil objective and images were taken with a CCD digital camera.

### Dye uptake

N2A cells were transfected with pIRES2EGFP2 clones for dye uptake assays with neurobiotin (NB, 287 Da, +1 charge, Vector Labs, USA). 24 h after transfection, cells were washed with PBS and incubated with Ca^+2^ free medium or medium with physiological calcium concentrations for 20 min at 37 °C. Cells were then incubated with 0.5 mg/ml NB for 20 min at 37 °C [19]. Next, cells were washed with PBS containing 3,2 mM CaCl_2_ three times for 10 min and were fixed with 4 % PFA for 20 min at room temperature. Subsequently, fixed cells were permeabilized with 0.1 % Triton-X 100 for 10 min, blocked with 3 % BSA-0.1 % TritonX-100 for 15 min and incubated with tetra-methyl rhodamine isothiocyanate (TRIT-C) conjugated streptavidin (1:1000 dilution, Pierce, USA) for 30 min at RT in dark. After washing with PBS three times for 10 min, images were acquired with a fluorescence microscope (IX71, Olympus, Japan) using the same exposure times. For carbenoxolone (CBX) treatment, cells were initially incubated with calcium free medium containing 100 μM CBX for 20 min and then NB in the same medium was applied to the cells as explained above.

Image analysis for signal intensity determination was performed with ImageJ (NIH, USA) program. During image analysis, after subtracting the background in merged images of red and green channels, the same parameters were applied to threshold the images for the measurement of red signal intensities of fluorescence only in GFP positive cells.

### Measurement of Intracellular calcium levels

Flow cytometry was used for the measurement of internal calcium content with Fluo-3 AM Ca^+2^ indicator (Invitrogen, USA) [18]. N2A cells (5x10^5^) were plated onto 6 well plates and transfected with pCS2+ clones. Cells were washed with Ca^+2^ free PBS or physiological medium twice for 5 min and incubated with 5 μM Fluo-3 AM at 37 °C for 30 min. Then, cells were washed with PBS, trypsinized and resuspended in divalent cation free Hank’s Balanced Salt Solution (HBSS) [44] and analyzed with FACSCANTO (BD Biosciences, USA).

### Colocalization analysis

The determination of colocalization of Cx26 and the Golgi apparatus marker, golgin-97, was carried on by using Fiji Coloc 2 plug-in. Costes’ colocalization coefficient was calculated that compares a pair of images from different fluorescent channels and quantifies the statistical significance of colocalization [45].

### Statistical analysis

All results were expressed as mean (± standard deviation). For colocalization quantification, first the pixels in the green and red channels were correlated to calculate a Pearson’s coefficient (PC) and then pixels in green channel were randomized and correlated with the original red channel for 200 times. PCs from 200 perturbations were compared with the original PC of non-randomized image and if original PC is higher than 95 %, a significant colocalization was accepted for the image. Groups were compared using ANOVA followed by Tukey’s HSD for all results. Colocalization data that only have positive or negative results were compared using Kruskal-Wallis rank sum test followed by Mann–Whitney test. Comparison of samples in the absence and the presence of CBX was done by using Student’s *t*-test. Statistical significance was considered for *p* < 0.05.

## Availability of data and materials

All the supporting data are included as additional files.

## Additional file

Additional file 1:
**Raw data for the following experiments: Cx26-golgin97 colocalization, neurobiotin uptake assay under physiological condition, neurobiotin uptake assay without calcium and in the presence of carbenexolone (CBX) and intracellular calcium content experiments.** (XLSX 25 kb)
